# Evaluation of a Patient Safety Advisory Among Inpatients—A Mixed Methods Study

**DOI:** 10.1111/scs.70034

**Published:** 2025-05-30

**Authors:** Margareta Bånnsgård, My Engström, Caterina Finizia, Johanna Moreno, Bojan Tubic

**Affiliations:** ^1^ Department of Otorhinolaryngology, Head and Neck Surgery, Institute of Clinical Sciences, Sahlgrenska Academy University of Gothenburg Gothenburg Sweden; ^2^ Institute of Health and Care Sciences, Sahlgrenska Academy University of Gothenburg Gothenburg Sweden; ^3^ Department of Surgery Sahlgrenska University Hospital, Region Västra Götaland Gothenburg Sweden; ^4^ Department of Research, Development, Education and Innovation Sahlgrenska University Hospital, Region Västra Götaland Gothenburg Sweden; ^5^ Department of Plastic Surgery, Institute of Clinical Sciences, Sahlgrenska Academy University of Gothenburg Gothenburg Sweden; ^6^ Department of Plastic Surgery Sahlgrenska University Hospital Gothenburg Sweden

**Keywords:** adverse events, patient education, patient involvement, patient participation, patient safety

## Abstract

**Objective:**

The purpose of this study was to examine patients' experiences with an educational leaflet containing information about how patients can participate in their care and keep themselves safe during their hospital stay.

**Methods:**

Patients from different wards at Sahlgrenska University Hospital and Skaraborg Hospital in Sweden were given the patient safety advisory during their hospital stay (*n* = 456). Patients (*n* = 219) completed a structured telephone interview 2–8 weeks after their discharge.

**Results:**

137 patients read the safety advisory during their stay and 82 patients did not. More patients who read the safety advisory stated that they received discharge instructions than patients who did not read it. The patients were overall positive towards the safety advisory. The advisory seemed to function as a reminder as the majority already knew the information. One quarter of participants reported that they changed their behaviour based on the safety advisory.

**Conclusions:**

The safety advisory seemed to promote behavioural change as 25% stated that they altered their actions after reading it. One challenge when advising patients about patient safety is optimising how the information is given, as more than a third of the participants did not read the safety advisory despite receiving it. This study indicates that the educational leaflet could promote safety behavioural change among inpatients.

## 1 Introduction

Adverse events were estimated to occur in approximately 11% of all hospitalisations in Sweden in 2020, and 60% of these were considered to be preventable, according to a systematic review of 8513 medical records conducted by the Swedish Association of Local Authorities and Regions [1]. This reported incidence of adverse events is comparable to studies conducted in other countries, as summarised in a systematic review that included a total of 74,485 patients and in which the incidence of in‐hospital adverse events was reported to be 9%, with a preventability of 43% [2]. An adverse event has been defined in the Harvard Medical Practice Study as an injury that was caused by medical management rather than the underlying disease and prolonged the hospitalisation, produced a disability at the time of discharge, or both [3]. The most common adverse events include healthcare‐associated infections, surgical complications, pressure ulcers, medication errors, urinary retention and falls [1].

One approach to reducing adverse events is to increase patient participation in care. Patient participation in care, also referred to as patient engagement or patient involvement, is a fundamental and important strategy to improve the quality of care [4].

Patients with high participation in their care are less likely to experience adverse events [5]. The importance of patient engagement has been recognised by the World Health Organisation in their initiative, ‘World Alliance for Patient Safety’, which was launched in 2004 [6]. The second action area in the initiative focused on patient involvement and sought to mobilise and empower patients through different strategies. For example, the Speak Up campaign enables patients to engage in their care through educational materials [7]. Building on earlier initiatives World Health Organisation introduced the Global Patient Safety Action Plan 2021–2030, which identifies patient and family as a strategic objective to enhance safety and reduce harm in healthcare settings [8].

Previous studies have indicated that patients are willing to participate in safety‐related behaviours but there is so far relatively limited evidence on their success in affecting behaviour change [9, 10, 11]. One approach to increasing patient participation in care is through the use of educational material. A previous study demonstrated that written and video‐based material together with a one‐on‐one follow‐up with a physiotherapist decreased the rate of falls in patients with intact cognition [12]. Another study examined the implementation of a patient safety programme consisting of an information leaflet, education for nurses and registration of adverse events, and found that the intervention group developed fewer adverse events than controls [13].

At Sahlgrenska University Hospital in Sweden, a patient safety leaflet was introduced in 2017 with the aim to increase patient participation and hopefully decrease adverse events. At Guy's and St. Thomas NHS Foundation Trust in England, a patient safety advisory was created in 2012 with the intention to increase patient safety. The idea arose from the aircraft safety card found on board airplanes. After obtaining approval from Guy's and St. Thomas' NHS Foundation Trust, the regional council Skåne in southern Sweden translated the advisory into Swedish in 2017 [14].

In 2017, Sahlgrenska University Hospital in the regional council, Region Västra Götaland, also obtained permission to modify the Swedish version of the safety advisory. The version used in Region Västra Götaland is called, ‘Your Safety at the Hospital’, and is evaluated in the current study (Appendix S2). It has been further developed and is now available in 10 different languages, and there is also one version aimed at paediatric patients and one directed towards psychiatric patients.

The patient safety advisory contains eight sections with information about different aspects of patient safety. It urges the patient to tell staff about any allergies they may have and which medicines they are taking. The safety advisory also encourages the patient to ask about their medications, care and treatment. It promotes behaviours such as standing up slowly and using non‐slip socks to decrease the risk of fall injuries, staying mobile to prevent blood clots, using hand sanitizer to prevent infections and changing position frequently to avoid pressure ulcers. It notifies them of which information they can expect to receive at the time of discharge, including discharge instructions. Discharge instructions are given to each patient at Sahlgrenska University Hospital and Skaraborg Hospital as part of the discharge routine and contain information about why the patient was admitted, information about the hospital stay, the plan ahead and a medication list.

This study aimed to evaluate, through structured interviews, patients' experience of the patient safety advisory, ‘Your safety at the hospital’.

## 2 Methods

### 2.1 Participants

**FIGURE 1:**
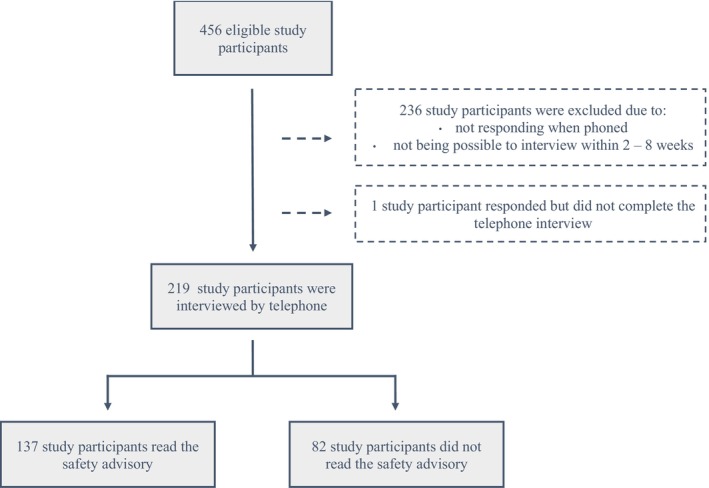
Flow chart describing the dropout and distribution of study participants.

A total of 456 patients were recruited for the study from different wards at Sahlgrenska University Hospital and Skaraborg Hospital. Inclusion criteria included being 18 years of age or older and knowledge of Swedish at a level where the participant could autonomously answer a questionnaire and participate in an interview. Patients with cognitive impairment were excluded from the study. Out of the 456 eligible study participants, 219 patients were interviewed by telephone (Figure 1). Potential participants were given both oral and written information about the study. Patients who agreed to participate gave their written consent. Ethical approval was obtained from the Ethical Review Board in Gothenburg (reference number 448‐17) prior to data collection.

### 2.2 Data Collection

Patients were recruited from 17 different medical and surgical wards at two different hospitals over a period from October 2017 to October 2020. All patients were given the safety advisory on the hospital ward together with oral information from different healthcare personnel working at the wards, e.g., nurses and assistant nurses. The intervention was presented to the healthcare personnel working at the wards and pocket cards (Appendix S1) containing the same information as the patient safety advisory were given to the hospital staff to facilitate and standardise the oral information. During their admission, the patients also responded to three questionnaires which are not evaluated in this study.

Between 2 and 8 weeks after being discharged from the hospital, the patients were contacted by telephone and asked to participate in a structured telephone interview. The respondents were asked demographic questions and about their stay at the hospital. They were also asked if they had suffered from different adverse events during their stay at the hospital.

Finally, they were asked if they had read the patient safety advisory, and those who had were asked further questions about their opinion on it. The questions in the structured interview were asked according to an interview guide (Appendix S3). The interview scripts were made from notes taken during the interview. The interviews were conducted by two of the researchers in Swedish and translated into English.

### 2.3 Data Analysis

This study was a mixed methods evaluation, with a focus on quantitative analysis. Statistical analyses were performed with IBM SPSS Statistics version 29 (New York, USA). Quantitative analysis was used to compare three different self‐reported adverse events (falls, nosocomial infections and pressure ulcers) between the group which read and the group which did not read the patient safety information. The rate of adverse events was also compared based on age and type of hospital ward (medical or surgical). The difference in the number of patients who received discharge information was also analysed between the group which read and did not read the safety advisory.

Chi‐square test, Fisher's exact test and Mantel–Haenszel test were used to test for statistically significant associations between categorical variables. Mann Whitney *U* test was used to compare differences between the variables, age, length of stay at the hospital and days from discharge until telephone interview.

Further qualitative analysis was conducted regarding the open‐ended questions about the safety advisory. Analysis of the free‐text responses was done through a summative approach to qualitative content analysis [15]. The analysis commenced with familiarisation with the data through re‐reading the notes. Data was first organised by clustering similar meaning units into categories. Identified categories were then combined into themes, see Table 1 for examples. Each response in every category was quantified.

**TABLE 1:** Example of diagram illustrating how data from telephone interviews were clustered when sharing similar meaning/characteristics.

Meaning units	Categories	Themes
Good summary about what's important to think about when you're admitted to the hospital	Positive characteristics	Overall impression
Don't have much experience from hospitals so yes, it was good	New and useful information	
Didn't apply to me because I knew I was only there for the day	Not relevant	

Author (MB) performed the initial analysis by organising similar meaning units into categories. The initial analysis was reviewed by author (ME), who independently examined the categories and themes, and suggested revisions. The preliminary categories and themes were then reflected upon and discussed with all the authors until consensus was achieved.

## 3 Results

### 3.1 Characteristics of Interviewed Patients

Baseline patient characteristics are presented in Table 2. A total of 219 patients were included in the study, with a mean age of 66 years (SD ± 14.4). 48% were men and 52% were women. 27% lived alone, whereas 67% lived together with someone and 6% were in a relationship but did not live with their partner. The mean time from discharge until the telephone interview was 36 days (SD ± 11.3). There were statistically significant differences between the groups who read or did not read the patient safety advisory regarding age, housing situation and depending on whether the patients were cared for in a medical or surgical ward (Table 2).

**TABLE 2:** Baseline patient characteristics (*n* = 219) for patients who did or did not read the patient safety advisory.

Description	Read safety advisory (*n* = 137)	Did not read safety advisory (*n* = 82)	*p*
**Age**			
Mean (SD)	64 (14.1)	70 (14.2)	< 0.001^a^
Age range (min − max)	25–92	20–93	
**Gender**			
Female	76 (55%)	38 (46%)	0.210^b^
Male	61 (45%)	44 (54%)	
**Housing situation**			
Living alone	29 (21%)	31 (38%)	0.002^c^
Living apart together	5 (4%)	8 (10%)	
Cohabitation	103 (75%)	43 (52%)	
**Length of hospital stay in days**			
Mean (SD)	5 (5.7)	5 (6.9)	0.254^a^
Range (min − max)	1–30	1–35	
**Care in medical or surgical ward**			
Medical ward	46 (34%)	48 (58%)	< 0.001^b^
Surgical ward	91 (66%)	34 (42%)	
**Days from discharge until telephone interview**			
Mean (SD)	38 (11.0)	35 (11.6)	0.076^a^
Range (min − max)	17–56	14–56	

^a^ Mann–Whitney *U* test.

^b^ Fishers exact test.

^c^ Chi‐square test.

### 3.2 Comparison of Self‐Reported Adverse Events

Seven participants reported that they had fallen during their stay at the hospital, while 22 patients stated that they had acquired an infection during their hospital stay. The most common infections were pneumonia, urinary tract infections, catheter‐associated infections, infections related to peritoneal dialysis, sepsis and infections of unknown origin. One participant developed a pressure ulcer while at the hospital. Those who suffered from an adverse event were significantly older than those who did not (*p* = 0.042). There was no statistically significant difference between the incidence of infections if the patient was treated in a medical or surgical ward (*p* = 0.264), nor in the different adverse events when comparing the group which had read the safety advisory to the group which had not read it (*p* = 0.714, *p* = 817, *p* = 1.000).

### 3.3 Discharge Instructions

The group which had not read the safety advisory more often stated they had not received or did not remember receiving discharge instructions compared to the group which had read it (*p* < 0.001, Table 3).

**TABLE 3:** Opinion on discharge instructions (*n* = 219), for patients who did or did not read the safety advisory.

	Read safety advisory (*n* = 137)	Did not read safety advisory (*n* = 82)	*p*
**Did you receive your healthcare and medication journal when discharged?**			
Yes	108 (79%)	45 (55%)	< 0.001^a^
No	24 (17%)	30 (37%)	
Did not remember	5 (4%)	7 (8%)	
**If yes, what did you think of it?**			
Positive	86 (80%)	35 (78%)	0.946^b^
Neutral/No opinion	12 (11%)	7 (15%)	
Negative	10 (9%)	3 (7%)	

^a^ Chi‐square test.

^b^ Mantel–Haenszel test.

Among those who received their discharge instructions, the majority (79%) were positive towards it. Several participants stated that the instructions were clear and easy to understand. Quite a few participants commented that it was good to receive written information and to have the possibility to go back later and read about their hospital stay. A 57‐year‐old participant shared, ‘There was a lot of information at once [when being discharged] so it's great being able to go back and read it’. The most common negative opinions were that the discharge instructions were too short and that information had been left out.

### 3.4 Opinion on Patient Safety Advisory

Four themes emerged from the telephone interviews: Overall impression, reminder, delivering information and increased involvement. These themes arose from 11 different categories. See Table 4 for a summary of the categories and themes.

**TABLE 4:** Summary of themes (bold text, grey boxes) and categories (white boxes) that emerged from the content analysis of the telephone interviews.

Overall impression	The safety advisory seemed to act as a reminder	Ways to facilitate delivery of information	Patient involvement increased to some extent
Positive characteristics	Nothing new	Digital information	Change in behaviour
Useful information	Memory refresher	Combining with oral information	Receiving information too late
Appreciated but not relevant for everyone	Already acting according to advice		Being too sick

### 3.5 Overall Impression

Most participants (83%) had a positive impression of the patient safety advisory. A commonly expressed opinion was that it was good to receive the information. Regarding its contents, several stated that it was easy to understand and informative. A 31‐year‐old participant shared, ‘Good summary about what's important to think about when you're admitted to the hospital’. Another 72‐year‐old participant added that the safety advisory was, ‘Informative and good. Adequate amount of information’.

Various participants expressed that although they appreciated the safety advisory, the information was not applicable for them. A 56‐year‐old participant stated, ‘Read it all, good to know, but nothing felt relevant to me then and there. But if it had I could've gone back to read it’. Similarly, a 57‐year‐old participant said, ‘Especially important if you're there for a longer period. But of course, there's a risk of falling even if you're only there for one day’.

### 3.6 The Safety Advisory Seemed to Act as a Reminder

A considerable amount of the participants (63%) expressed that the safety advisory did not contain any new information for them. Some perceived the information as obvious. A few stated that they knew it because they had been admitted before, or because they worked in healthcare. A 66‐year‐old participant said, ‘Nothing new, have been admitted in the past so I already knew it’.

Even though the information was known for the majority of interviewed participants, it was still appreciated as a reminder. A 65‐year‐old participant commented, ‘Obvious stuff but it deserves a reminder’. A 54‐year‐old participant added, ‘Can't mention anything specific that was new, but still good to hear it again. It's like when you're flying and the information is repeated over and over again, and that's good’.

Although some changed their behaviour after reading the safety advisory, the majority reported that they did not do so after reading the safety advisory. Quite a few stated that since they were aware of the information, they already acted accordingly to the advice included in the advisory. One 83‐year‐old participant shared, ‘[I] am being careful anyways’.

### 3.7 Ways to Facilitate Delivery of Information

One 48‐year‐old study participant mentioned that she would have liked to receive the information on the safety advisory digitally, ‘Also [I] would have wished to receive it by email. Easier for you to update digital information as well’. Another 48‐year‐old participant stated that she preferred receiving oral information instead of written information, ‘Asked around instead, suits me better than reading’. The degree to which the patients received oral information in combination with the written information seemed to vary since another 44‐year‐old participant commented, ‘It was also repeated by the nurses so they were really thorough about it’.

### 3.8 Patient Involvement Increased to Some Extent

A quarter of the participants (25%) stated that they changed their behaviour based on the patient safety advisory. The most common answers to how the participants changed their behaviour were being extra careful when standing up and using non‐slip socks to avoid falls. Other ways the participants increased their involvement were by using hand sanitizer more to prevent infections and moving as much as possible to avoid blood clots. As an example, a 60‐year‐old participant commented, ‘Was there for such a short time but tried to not just lay in bed all the time. Extra hygiene too, wiped off stuff that was borrowed from each other which you probably wouldn't have thought about otherwise’.

One barrier for involvement in the safety behaviours that was mentioned by a participant was that he received the information too late. In this case, the 58‐year‐old participant pointed out that he ‘Received it the last day so it wasn't relevant then’. Another hindrance to participating in the safety behaviours was being too disabled to do so, as one 77‐year‐old participant commented, ‘Was in pretty bad condition. Needed help to reposition in bed. And help to get up’.

## 4 Discussion and Conclusion

### 4.1 Discussion

The group which did not read the patient safety advisory was significantly older and lived alone to a greater extent compared with the group which read it. Health issues are more common among elderly people, and elderly living alone have reported a higher prevalence of poor health and falls compared to those living together with someone [16]. The rates of experiencing adverse events have previously been demonstrated to increase with age [3] and increased age has previously been identified as a factor associated with less involvement [17]. This study indicates that those of older age and living alone did not read the safety advisory to the same extent as those of younger age and living with someone. Because this group is at risk of poor health and adverse events, our results indicate that it would be important to direct more attention to them when informing about patient safety.

Among those treated in surgical wards, more patients read the safety advisory. To our knowledge, no previous studies investigate the differences between medical and surgical wards in the implementation of patient education interventions. One can speculate that the patient safety information might be easier to implement among the health‐care personnel, as structured information about surgical treatments is commonly provided and might also include information about patient safety, for example the importance of preventing blood clots [18, 19].

Another finding in this study is that among those who read the safety advisory, more participants reported that they received their discharge instructions than those who did not read it. Materials focusing on patient actions have been suggested as an intervention to improve comprehension of discharge instructions [20]. It is possible that the safety advisory contributed to an increased awareness of what the discharge instructions are.

More than a third of the study participants did not read the safety advisory. There could be several reasons for this. For example, factors such as information overload, patients' physical or emotional state during hospitalisation, or variability in how the advisory was introduced by staff [21]. Health literacy is an important concept to consider when discussing patient involvement, as low health literacy can hinder patient understanding or prioritising the safety advisory. One of the main obstacles to patient participation is low health literacy and lack of knowledge of the subject [22]. Providing sufficient knowledge is essential, as previous studies have established that informed patients are more willing to participate in patient safety work [23].

To enhance understanding and engagement, the leaflet could be complemented with educational videos and personalised information from healthcare personnel. Combining written with oral information has been demonstrated to be better remembered and lead to better treatment adherence [24], and the extent to which the participants received the safety advisory together with oral information seemed to vary. Therefore, providing structured oral information together with the safety advisory could be a strategy to improve adherence.

Using audiovisual media is another communication method that has the potential to convey patient safety information effectively. A previous study demonstrated that an educational programme consisting of a motion graphic video improved inpatients' knowledge and awareness of patient safety compared to written information [25]. The same information evaluated in the current safety advisory is also available in video format, which can serve as an appealing complement to conventional written information [26].

One quarter of those who read the safety advisory stated that they changed their involvement in patient safety work. These findings are in line with a previous study that concluded that an educational video and leaflet could increase patients' perceived comfort in participating in safety‐related behaviours [27]. There were several examples of how the patients increased their involvement; the most common was changing behaviour to prevent falls, infections and blood clots. The responses from the interviews suggested that the information communicated on the safety advisory promoted these changes in behaviour. This study did not find a lower incidence of adverse events among those who read the safety advisory, however, which was expected considering the relatively small sample size. Further investigation needs to be conducted to determine the effect of the safety advisory on the incidence of adverse events.

### 4.2 Strengths

The sample size is relatively large and includes patients from across multiple wards, which can be considered a strength in this study. The use of a mixed‐methods design is also a strength, as it combines quantitative data comparing the groups that read and did not read the safety advisory with qualitative insights, providing a deeper understanding of the experiences of patients who read the safety advisory.

### 4.3 Limitations

The data is self‐reported and gathered sometime after the intervention, which could be a potential limitation as it may result in recall bias, affecting the patients' memory of the safety advisory. The extended duration of the data collection may have led to inconsistencies in how the intervention was delivered. The safety advisory was also distributed by multiple healthcare personnel across several hospital wards which is a potential limitation as interruptions or limited time for patient education might have affected how the information was given. In the qualitative part of this study, efforts were made to ensure trustworthiness through reviewing the analysis and continuous dialogue among the authors. However, qualitative research is still influenced by the researchers' preconceptions, which remains a consideration.

## 5 Conclusion

This study aimed to examine how the patients experienced the safety advisory, ‘Your Safety at the Hospital’, through structured telephone interviews. One third of the study participants stated they had not read the patient safety advisory during the hospital stay, and this group was significantly older and lived alone to a greater extent than those who read it. Among those who read it, the patient safety advisory was generally viewed positively and seemed to function as a reminder about safety to the patients, who reported already knowing much of the included information. The safety advisory also may have encouraged changes in behaviour among participants, as 25% stated they altered their actions in different ways after reading it.

### 5.1 Practice Implications

Adverse events contribute to a vast amount of patient suffering as well as economic costs. There are no previous studies evaluating the how the patient safety advisory ‘Your safety at the hospital’ is perceived by the patients. Our findings indicate that this educational leaflet could promote safety behavioural change among inpatients. Since older adults, those living alone and patients treated on medical wards did not read the safety advisory to the same extent, further research could focus on developing strategies for tailored education aimed at these groups.

## Author Contributions

C.F. and B.T. devised the project and the main conceptual idea. J.M. and B.T. collected the data. M.B. analysed the data with aid from M.E. M.B. wrote the manuscript with support from M.E., C.F. and B.T. All authors discussed the results and contributed to the final manuscript.

## Ethics Statement

Ethical approval was obtained from the Ethical Review Board in Gothenburg (reference number 448‐17) prior to data collection.

## Conflicts of Interest

The authors declare no conflicts of interest.

## Supporting information


Appendix S1.

Appendix S2.

Appendix S3.

## Data Availability

The data that support the findings of this study are available on request from the corresponding author.
