# Supplementary material for: Evaluation of a Patient Safety Advisory Among Inpatients—A Mixed Methods Study
Source: Scand J Caring Sci. 2025 May 30;39(2):e70034. doi: 10.1111/scs.70034 (PMC12125419; doi:10.1111/scs.70034)
Supplement: Supplementary file 1 — Appendix S1. [file SCS-39-0-s001.pdf]

Oral patient safety information SU

- To be used when presenting the safety advisory ”Your safety at the hospital”

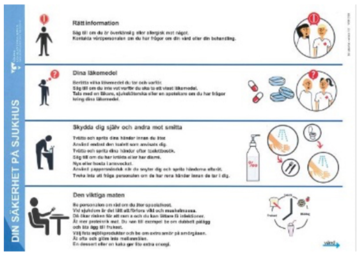

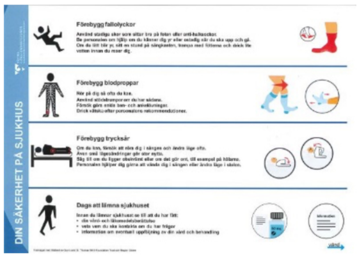

- Do you know if you are oversensitive to any medications or anything else?
- We need to know what medicines you take any why. Please ask us if you have questions about your medicines or about your care and treatment.
- During your hospitalization it is extra important to prevent infections from spreading between each other. Therefore keep in mind to both wash and rinse your hands with alcohol after you have used the toilet and before meals.
- Is there something you cannot eat? During hospitalization it is important to get enough nutrition since this helps wounds to heal better for example. Try to eat and drink regularly during the day. During your time at the hospital it can be good to ask for double sandwich fillings and higher fat dairy products, and also to eat snacks between meals for a little extra energy.

If the patient is planned for surgery and should fast;
tell the patient that the part about the food applies after surgery

Sahlgrenska Universitetssjukhuset

Developed by the R&D-department section 5 and the research group Your safety at the hospital

Oral patient safety information SU

- Be careful when standing up. If you feel dizzy, sit for a while on the edge of the bed and walk with your feet in the air. Please ask the us for help if you feel unstable so you do not fall. It is easy to slip on the floor, and to avoid it you need sturdy shoes or shoes with rubber soles. If you do not have such shoes, we can give you anti-slip socks.
- The risk of getting blood clots increases when you are still. Move as often as you can. It can be a short walk in the corridor or walking to the dining area to have your meals. If it is hard for you to walk, try to sit up in the bed or in an armchair and move your legs and ankles a little extra.
- By moving in the bed and changing your position you are decreasing the risk of bed sores. If you in sitting or lying in an uncomfortable position and cannot change position by yourself, it is important to tell us so we can help you change the position.
- When it is time to leave the hospital you will receive information about plan ahead and who you can contact if you have any questions. You will find this information in the healthcare and medication journal which you will receive from the hospital, or separately from the hospital ward.
- Please read the safety advisory “Your safety at the hospital” for more information on how you can prevent adverse events. We want your hospitalization time to be as good and safe as possible.

Also recommend the patient to watch the film ”Your safety at the hospital” if it is available on the ward.
