# Supplementary material for: Evaluation of a Patient Safety Advisory Among Inpatients—A Mixed Methods Study
Source: Scand J Caring Sci. 2025 May 30;39(2):e70034. doi: 10.1111/scs.70034 (PMC12125419; doi:10.1111/scs.70034)
Supplement: Supplementary file 2 — Appendix S2. [file SCS-39-0-s002.pdf]

Engelska

YOUR SAFETY IN HOSPITAL

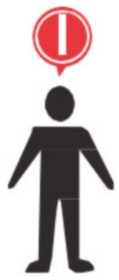

Correct information

Tell us if you are over-sensitive or allergic to anything.
Ask the medical staff if you have questions about your care or your treatment.

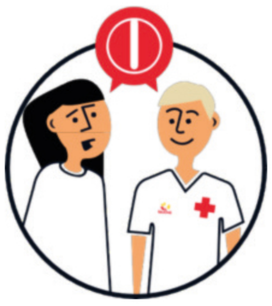

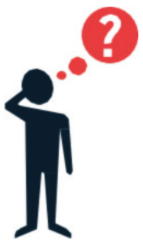

Your medicines

Tell us what medicines you take and what for.
Tell us if you don't know why you must take a particular medicine.
Talk with a doctor, a nurse or a pharmacist if you have questions about your medicines.

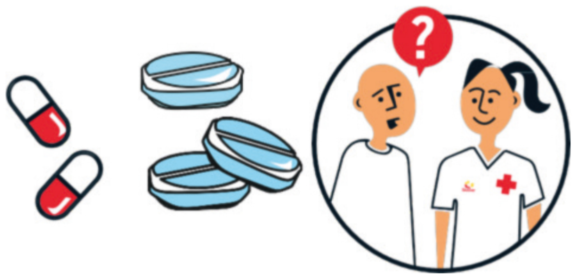

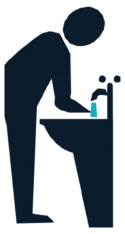

Protect yourself and others against infections

Wash and rinse your hands with alcohol before you eat.
Only use the toilet shown to you. Wash and rinse your hands with alcohol after using the toilet. Tell us if you have vomited or have diarrhoea.
Sneeze or cough into your arm.
Use a paper handkerchief when you blow your nose and rinse your hands with alcohol afterwards.
Do not hesitate to ask the staff if they have clean hands before they touch you.

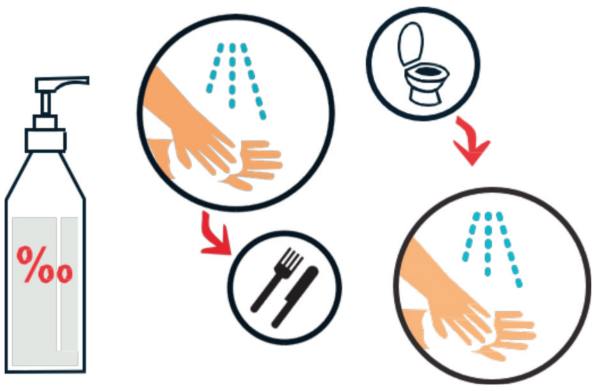

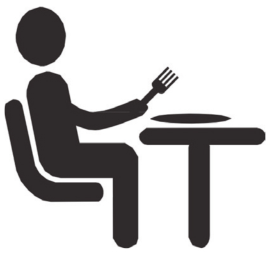

The importance of food

Ask the staff for advice if you have a special diet. It is easy to lose weight and muscle mass when you are ill. This increases the risk of falling and you can get infections more easily.
Eat more high-protein food. For example, you can ask for double sandwich fillings and eat eggs for breakfast. Choose higher fat dairy products and ask for extra butter on your sandwiches. Eat often and do not forget to eat snacks between meals. A dessert or a piece of cake will give you a little extra energy.

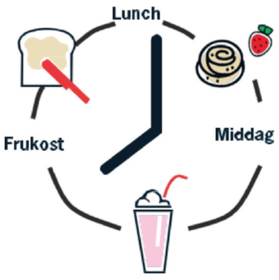

Please turn over

YOUR SAFETY IN HOSPITAL

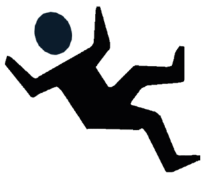

Preventing falls

Use sturdy shoes that fit your feet well, or anti-slip socks.
Ask the staff for help if you feel dizzy or unstable when you get up to walk. If you easily get dizzy, sit for a while on the edge of the bed, walk with your feet in the air and drink some water before you get up.

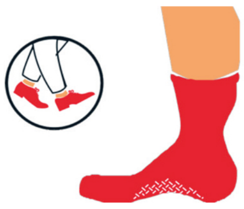

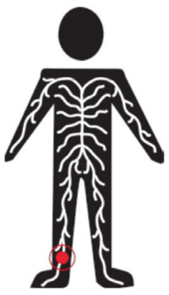

Preventing blood clots

Move as often as you can.
Use compression socks if you have them.
Try to do simple leg and ankle exercises.
Drink liquids as recommended by the staff.

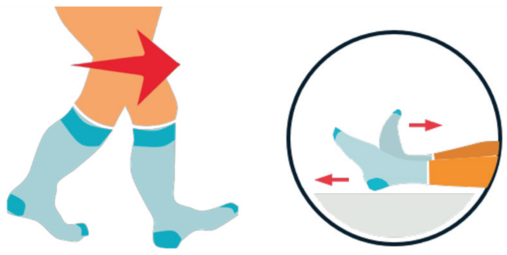

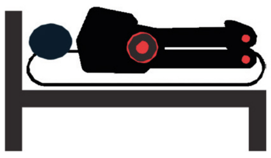

Preventing bed sores

If you can, try to move in the bed and change your position often.
Even small changes in position help.
Tell us if you are uncomfortable lying down or if it hurts, such as on your heels.
The staff will be happy to help you turn over in bed or change your position in the chair.

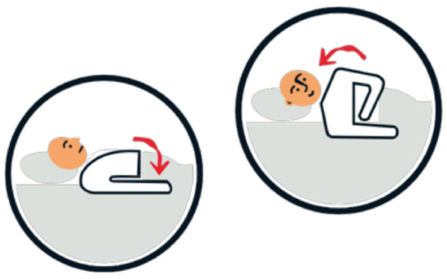

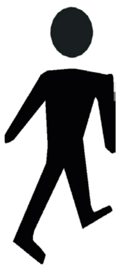

Time to leave the hospital

Before you leave the hospital, make sure you:
- obtain your healthcare and medication journal
- know who to contact if you have questions
- information about following up your care and treatment

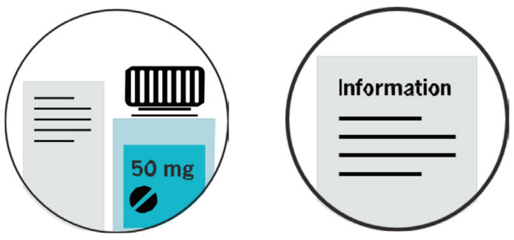

Please turn over

Framtaget av Sahlgrenska Universitetssjukhuset med tillstånd av Guy´s and St. Thomas NHS Foundation Trust och Region Skåne
