# Supplementary material for: Evaluation of a Patient Safety Advisory Among Inpatients—A Mixed Methods Study
Source: Scand J Caring Sci. 2025 May 30;39(2):e70034. doi: 10.1111/scs.70034 (PMC12125419; doi:10.1111/scs.70034)
Supplement: Supplementary file 3 — Appendix S3. [file SCS-39-0-s003.pdf]

Safety on ward

Appendix 3

Structured telephone interview guide:

1: Name and age

2: Do you live alone, with someone else or other option?

3: How long was your stay at the hospital?

4: How has it been since your discharge? Current need of any nursing care?

5: During your hospitalization did you experience any:

-Accidental falls? How many times?

- Health-care associated infections? If yes – what kind of infection

- Pressure ulcers? If yes – which part of the body?

6: Did you gain or lose weight during the hospital stay? How many kilograms? Weight at arrival? Weight at discharge? Height?

- If the study participant answered yes: Any known incident or possible causes?

7: Did you receive a discharge letter?

- If the study participant answered yes: Thoughts on the discharge letter?
- If the study participant answered no: Possible causes to not receiving a discharge letter?

Questions regarding the patient safety advisory: "Safety on ward"

1. Did you read the information on the patient safety advisory "Safety on ward"? Yes/No
2. What did you think about it? Alternatively, about the oral information (if only that was communicated (patient group 1) about patient safety that you were given by the healthcare workers.

   ____
3. Was there any new information on the safety advisory? Yes/No
   If the study participant answered yes: which information was new?

   ____
4. Was any information on the safety advisory beneficial/useful? Yes/No
   If the study participant answered yes: Which part/information was useful?

   ____
5. Was the safety advisory most useful/beneficial at the ward or at discharge?
   ☐ At the ward ☐ Before discharge ☐ Equally useful/beneficial at ward/before discharge
6. Did the patient safety advisory change your involvement in the patient safety work? If the study participant answered yes: What have you changed?
7. Are you satisfied with your stay at the hospital? What was good? What can be improved? How can it be improved?

8. Do you think that you received enough information to be involved in your own care? What was good? What can be improved? How can it be improved?

9. Where would estimate your current state of health on a scale from 0-100? Where zero is the worst possible state of health and 100 is the best possible state of health (Self-assessed health).

10. Other comments regarding this study?
